# Identification and validation of biomarkers related to lysine β-hydroxybutyrylation in chronic obstructive pulmonary disease based on transcriptomics data

**DOI:** 10.1186/s41065-026-00682-x

**Published:** 2026-04-24

**Authors:** Jing Lei, Jing Zhang, Linfei Yang, Yubao Chen

**Affiliations:** Department of Respiratory and Critical Care Medicine, Nanjing First Hospital, Nanjing Medical University, 68 Changle Road, Qinhuai District, Nanjing, Jiangsu 210000 China

**Keywords:** Machine learning, Immune infiltration, Molecular docking, Nomogram model

## Abstract

**Background:**

Chronic obstructive pulmonary disease (COPD) involves progressive lung inflammation and tissue destruction. Lysine β-hydroxybutyrylation (Kbhb) is linked to COPD. This study aimed to identify Kbhb-related biomarkers for COPD to aid therapeutic development.

**Methods:**

COPD data and Kbhb-related genes (Kbhb-RGs) were obtained from public databases and literature. Candidate genes were identified by overlapping differentially expressed genes (DEGs) with Kbhb-RGs. Biomarkers were selected using machine learning. Diagnostic efficacy was evaluated via a nomogram. Functional enrichment, immune infiltration, drug prediction, and molecular docking analyses were performed. Biomarker expression was validated by reverse transcription quantitative polymerase chain reaction (RT-qPCR).

**Results:**

A total of 12 candidate genes were detected at the intersection of 765 DEGs and 1,493 Kbhb-RGs. Subsequently, POLD2 and OTUD7B were identified as biomarkers, and the expression of these 2 genes was found to be downregulated in COPD samples. The nomogram developed utilizing these biomarkers demonstrated a satisfactory capacity for differentiating among various sample types. Biomarkers were significantly enriched in transcriptional regulation and translation processes. Regulatory T cells displayed a significant positive linkage with OTUD7B (correlation (r) > 0.3, *P* < 0.05). Eosinophils were considerably negatively relevant to POLD2 and OTUD7B (*r* < -0.3, *P* < 0.05). Molecular docking studies demonstrated a strong binding affinity between biomarkers and estradiol. Compared with the control group, the expression levels of POLD2 and OTUD7B were significantly lower in the COPD group.

**Conclusions:**

This study identified POLD2 and OTUD7B as biomarkers for COPD, offering valuable insights that could support the development of targeted therapies.

**Supplementary Information:**

The online version contains supplementary material available at 10.1186/s41065-026-00682-x.

## Background

 Early diagnosis and treatment are crucial for improving prognosis and overall survival in COPD patients [1]. Currently, COPD diagnosis primarily relies on pulmonary function tests. Additional methods, including impulse oscillometry, chest radiography, magnetic resonance imaging, and computed tomography, have been developed to enhance diagnostic accuracy. However, owing to limitations in sensitivity and specificity, existing diagnostic methods remain insufficient for detecting early-stage COPD [2]. Early diagnosis and treatment are crucial for improving prognosis and overall survival in COPD patients [1]. Currently, COPD diagnosis primarily relies on pulmonary function tests. Additional methods, including impulse oscillometry, chest radiography, magnetic resonance imaging, and computed tomography, have been developed to enhance diagnostic accuracy. However, owing to limitations in sensitivity and specificity, existing diagnostic methods remain insufficient for detecting early-stage COPD [2]. Therefore, it is urgent to explore new biomarkers and understand their regulatory mechanisms in COPD for effective early diagnosis strategies to reduce the risk of COPD occurrence.

Histone modifications play crucial roles in epigenetic regulation, governing diverse chromatin-associated processes, including gene expression [3]. Recent studies have demonstrated that β-hydroxybutyrate (BHB) condenses with free coenzyme A (CoA) to form BHB-CoA, which serves as a high-energy donor for histone Kbhb [4]. Kbhb represents a newly identified post-translational modification that functionally links metabolic states to gene expression, enabling rapid cellular responses to environmental perturbations [5]. Acting as a molecular bridge between energy metabolism and epigenetic regulation, Kbhb participates in the modulation of gene transcription, inflammatory responses, and cell fate determination [6, 7]. Jiang et al. demonstrated that BHB dehydrogenase 1 mediates lung adenocarcinoma progression through the H3K9kbhb/leucine-rich repeat protein 31 axis [8]. Patients with COPD frequently exhibit disorders in energy metabolism and aberrant cellular stress responses, processes that may be regulated by Kbhb modifications. Nevertheless, the specific role of this regulatory mechanism in COPD remains incompletely understood. In recent years, numerous COPD-related hub genes (APP, FN1, SPP1, among others) have been successfully identified through transcriptomics-based bioinformatics analyses, with their expression patterns subsequently validated in cellular and clinical samples [9, 10]. Although these studies have established the feasibility of utilizing omics approaches to identify COPD molecular markers, no comprehensive investigation has yet been conducted from the perspective of Kbhb, a metabolic-associated epigenetic modification. Therefore, investigation of Kbhb-related biomarkers and their interactions with COPD pathogenesis may provide novel strategies for early diagnosis and therapeutic intervention.

In this study, we identified differentially expressed genes (DEGs) in COPD using publicly available transcriptomic data. DEGs were then intersected with Kbhb-RGs to identify candidate genes. Subsequently, machine learning algorithms and experimental validation were employed to confirm biomarkers. Finally, these biomarkers were characterized through drug prediction analysis, immune infiltration assessment, and functional enrichment analysis. These findings provide novel evidence supporting further investigation of Kbhb-mediated mechanisms in COPD and their potential clinical applications.

## Methods

### Data collection

The data of COPD were gained from the Gene Expression Omnibus (GEO) database. The GSE248493 dataset (platform: GPL18573) was employed as a training set, which included 25 COPD peripheral blood mononuclear cell (PBMC) and 12 control PBMC samples. The GSE94916 dataset (platform: GPL20844) served as a validation set, which comprised 6 COPD PBMC samples and 6 control PBMC samples. The GSE171541 dataset, based on the GPL24676 platform (Illumina NovaSeq 6000), comprised 9 samples, including 3 COPD samples and 6 normal lung tissue samples, and was primarily used for analysis at the single-cell level. In addition, a total of 1,493 Kbhb-RGs were obtained from the relevant literature [11] (Additional file 1). To systematically identify biomarkers associated with COPD, we employed a bioinformatics analysis workflow consistent with previous studies [12, 13]. First, DEGs were identified using transcriptome data from the GEO database. Subsequently, multiple machine learning algorithms were applied to perform dimensionality reduction and screening of candidate genes, thereby determining potential biomarkers with high confidence.

### Differential expression analysis

To identify DEGs between COPD and control groups, the analysis of DEGs was carried out by employing the DESeq2 package [14] (v 1.40.2) based on training set for both groups of samples (|log_2_Fold Change (FC)| > 0.5, *P* < 0.05). Moreover, the ggplot2 package (v 3.4.1) [15] was utilized to construct a volcano plot of DEGs, and the top 10 genes (sorted by P value from low to high) with significant up- or down-regulation were labeled, and then the heatmap of these genes was drawn using the ComplexHeatmap package (v 2.15.1) [16].

### Identification and functional enrichment of candidate genes

To identify the genes associated with Kbhb in COPD, the ggvenn package (v 0.1.10) [17] was employed to determine the intersection of DEGs and Kbhb-RGs. These genes were subsequently defined as candidate genes for further analysis. Subsequently, to explore the biological pathways of these genes and their functional enrichment, enrichment analysis of candidate genes was analyzed by Gene Ontology (GO) [molecular function (MF), biological process (BP) and cellular component (CC)] and Kyoto Encyclopedia of Genes and Genomes (KEGG) via the clusterProfiler package [18] (v 4.2.2) (*P* < 0.05).

### Machine learning and expression validation

In order to further refine the selection of candidate genes, Least Absolute Shrinkage and Selection Operator (LASSO), Support Vector Machine-Recursive Feature Elimination (SVM-RFE), Boruta, eXtreme Gradient Boosting (XGBoost), and Random Forest (RF) analyses were performed on the training set. The LASSO regression analysis was performed on the candidate genes using the glmnet package (v 4.1.8) [19]with an L1-regularized logistic regression model (elastic net mixing parameter α = 1). The optimal regularization parameter (lambda) was determined via 5-fold cross-validation using the cv.glmnet function, selecting the lambda.min value that minimized the cross-validation error. Genes with non-zero coefficients (excluding the intercept) under this optimal lambda were retained as LASSO genes. The SVM-RFE algorithm was implemented using the e1071 package (v 1.7.16) (https://cran.r-project.org/web/packages/e1071/) for feature selection, employing a radial basis function (RBF) kernel with the default penalty coefficient (C = 1). Feature importance was iteratively evaluated through 5-fold cross-validation, and the least important features were successively eliminated. The final optimal feature subset was selected as the one that yielded the minimum cross-validation error rate during this iterative process. Additionally, the Boruta algorithm was implemented using the Boruta R package (v 8.0.0) [20] with parameters set to a maximum of 100 iterations (maxRuns = 100) and a significance threshold of *P* = 0.01. The algorithm identifies robust features by calculating Z-scores of importance via random forests and comparing them with randomly generated shadow features, ultimately defining those classified as “Confirmed” as Boruta genes. The XGBoost analysis was conducted on candidate genes using the xgboost package (v 2.1.1.1) (https://xgboost.readthedocs.io/en/stable/R-package/index.html). The parameters of the classification tree were set as follows: max_depth = 2, eta = 0.3, and nrounds = 30. XGBoost genes were selected based on their importance scores and corresponding rankings. The importance scores of the candidate genes were calculated using the randomForest package (v 4.7.1.2) [21] with parameters set to 100 trees and mtry = 3. The genes were then ranked based on their importance scores in descending order. The optimal feature subset, designated as RF genes, was selected as the set of genes present when the out-of-bag (OOB) error rate reached its minimum.

Furthermore, the ggvenn package was applied to gain the intersection of genes identified by the 5 algorithms, which were defined as candidate biomarkers. This intersection strategy was designed to filter out potential spurious associations that might arise from small sample sizes through independent validation by multiple algorithms, thereby enhancing the robustness and reproducibility of the final biomarker screening results. An investigation was conducted to compare the expression of candidate biomarkers in the COPD and control groups, using the GSE248493 and GSE94916 datasets. The Wilcoxon test was employed to analyse the datasets (*P* < 0.05). Genes that exhibited stable expression in both datasets and showed significant differences were selected as biomarkers.

### Developing and assessing a nomogram

Using the training set, a nomogram model was formulated, underpinned by biomarkers, using the rms package (v 6.8.1) (https://CRAN.R-project.org/package=rms) to determine the likelihood of COPD occurrence. To appraise the forecasting precision of the nomogram, a calibration curve was generated by the regplot package [22] (v 1.1.0), and the Hosmer-Lemeshow (HL) test was executed to assess the fit between predicted values and actual outcomes (*P* > 0.05). The Receiver Operating Characteristic (ROC) curve was deployed to ascertain the effectiveness of the nomogram in a clinical setting via the pROC package (v 1.18.5) [23], with an area under the curve (AUC) > 0.7 considered a valuable reference for favorable diagnostic efficacy.

### GeneMANIA network and gene set enrichment analysis (GSEA)

In an effort to gain deeper insights into of the interactions between biomarkers and their associated biological functions, a gene co-expression network was constructed using the Gene Multiple Association Network Integration Algorithm (GeneMANIA) website. The GSEA was carried out with the objective of elucidating the biological functions of biomarkers throughout the progression of COPD. The reference gene set (c2.cp.v2024.1.Hs.symbols.gmt) was retrieved from the Molecular Signatures Database. The initial step was to calculate the Spearman correlation coefficients between each biomarker and each gene across all samples from the training set, using the psych package (v 2.2.9) [24]. Following that, the sorted genes were then arranged in descending order according to their respective Spearman correlation coefficients, with this ranking then being utilised as the gene set that was tested in further analyses. Subsequently, the GSEA was carried out employing the clusterProfiler package (v 4.2.2), with thresholds of |Normalized Enrichment Score| > 1, adj. *P* < 0.05.

### Immune infiltration analysis

To characterize the immune cell infiltration from COPD patients and healthy controls, the relative ratios of 22 types of infiltrating immune cells [25] were quantified using the CIBERSORT algorithm [26] (v 0.1.0) in the training set. Moreover, the Wilcoxon test was conducted to identify immune cells exhibiting notable disparities between the COPD and control groups (*P* < 0.05). Subsequently, the psych package was applied to analyze the correlations between the biomarkers and the significantly different immune cells (|cor| > 0.3, *P* < 0.05).

### Regulatory network construction

The 2 regulatory networks were constructed to investigate the molecular regulatory mechanisms of the biomarkers First, microRNAs (miRNAs) targeting the biomarkers were predicted via the miRDB database (https://mirdb.org/) and the miRWalk database (http://mirwalk.umm.uni-heidelberg.de/). Key miRNAs were ascertained by crisscrossing forecast miRNAs from both databases. In addition, the hTFtarget database (http://bioinfo.life.hust.edu.cn/hTFtarget) was employed to predict Transcription Factors (TFs) targeting the biomarkers. Based on the above results, TF-mRNA and miRNA-mRNA networks were constructed using Cytoscape software (v 3.8.2) [27].

### Potential drug prediction and molecular docking

In order to identify drugs that might interact with biomarkers, potential drug candidates targeting these genes were analyzed using the Drug Signature Database (https://www.dsigdb.org/). The results was visualized via Cytoscape software (v 3.8.2) to visualize the biomarker-drug interaction network. To determine the binding affinity between pharmaceutical agents and biomarkers, drugs targeting the same biomarkers were selected for molecular docking. The structure files of the drugs were sourced from the PubChem database, and the protein structures of the biomarkers were extracted from the Protein Data Bank. Afterward, molecular docking was conducted utilizing the AutoDockTools package [28] (v 1.5.7). The binding activity was deemed to be satisfactory if the affinity was lower than − 5.0 kcal/mol. The configuration of the receptor-ligand binding mode and the intermolecular forces were determined using PyMOL software [29] (v 3.1).

### Processing and annotation of single-cell RNA sequencing (scRNA-seq) data

First, the sequencing data in GSE171541 were filtered using the “Seurat” package (version 5.0.1) [30]. Performed quality control on the single-cell data, retaining cells that simultaneously satisfied the following conditions: unique molecular identifier (UMI) count (nCount_RNA) was between 500 and 100,000; the number of expressed genes (nFeature_RNA) was between 300 and 7,000; mitochondrial gene proportion (percent.mt) was below 10%. Additionally, genes expressed in fewer than three cells were removed. Then, the FindVariableFeatures function was employed to extract the top 2000 highly variable genes based on their correlation between mean and variance for subsequent analysis. Subsequently, a principal component analysis (PCA) was executed on samples in the dataset, and a permutation test for the null distribution was implemented employing the JackStraw function to display the percentage of variance of the top 20 principal components (PCs). At the same time, an inflection point plot of the change in standard deviation for the top 20 PCs was plotted. The PCs that contributed the most to the variance and had a p-value under 0.05 were selected for downstream analysis. After determining the PCs, unsupervised clustering analysis of the cells was conducted employing FindNeighbors and FindClusters functions (resolution = 0.1), and the cells were clustered into different clusters using Uniform Manifold Approximation and Projection (UMAP) clustering method. Subsequently, different cell types were annotated and compared using the “SingleR” (version 2.0.0) [31]and CellMarker database (http://117.50.127.228/CellMarker/). Simultaneously, the FeaturePlot function was used to visualize the expression distribution of biomarkers across different cell types in UMAP dimensionality reduction plots. Next, differences in cellular composition between the disease group and the control group were compared using the chi-square test based on cell type proportions.

### Reverse transcription quantitative polymerase chain reaction (RT-qPCR)

For this research, blood samples were collected from 5 COPD patients and 5 healthy controls at Nanjing First Hospital. These specimens were then utilized for RT-qPCR analysis. The research received ethical approval from KY20250811-KS-01. The primer sequences used for qPCR were listed in Additional file 2. To determine the relative quantification of biomarkers, the 2^−ΔΔCT^ approach was implemented. The RT-qPCR data were initially organized in Excel, then statistically analyzed and plotted using GraphPad Prism 10 software (*P* < 0.05).

### Statistical analysis

The R (v 4.3.1) was used for bioinformatics analysis. Discrepancies between groups were evaluated via the Wilcoxon rank-sum test. In the case of RT-qPCR analysis, inter-group comparisons were executed by means of the t-test. Notably, in our study, ***P < 0.001, **P < 0.01, *P < 0.05, and ns *P* > 0.05.

## Results

### The 12 candidate genes were associated with Kbhb in COPD

**Fig. 1:**
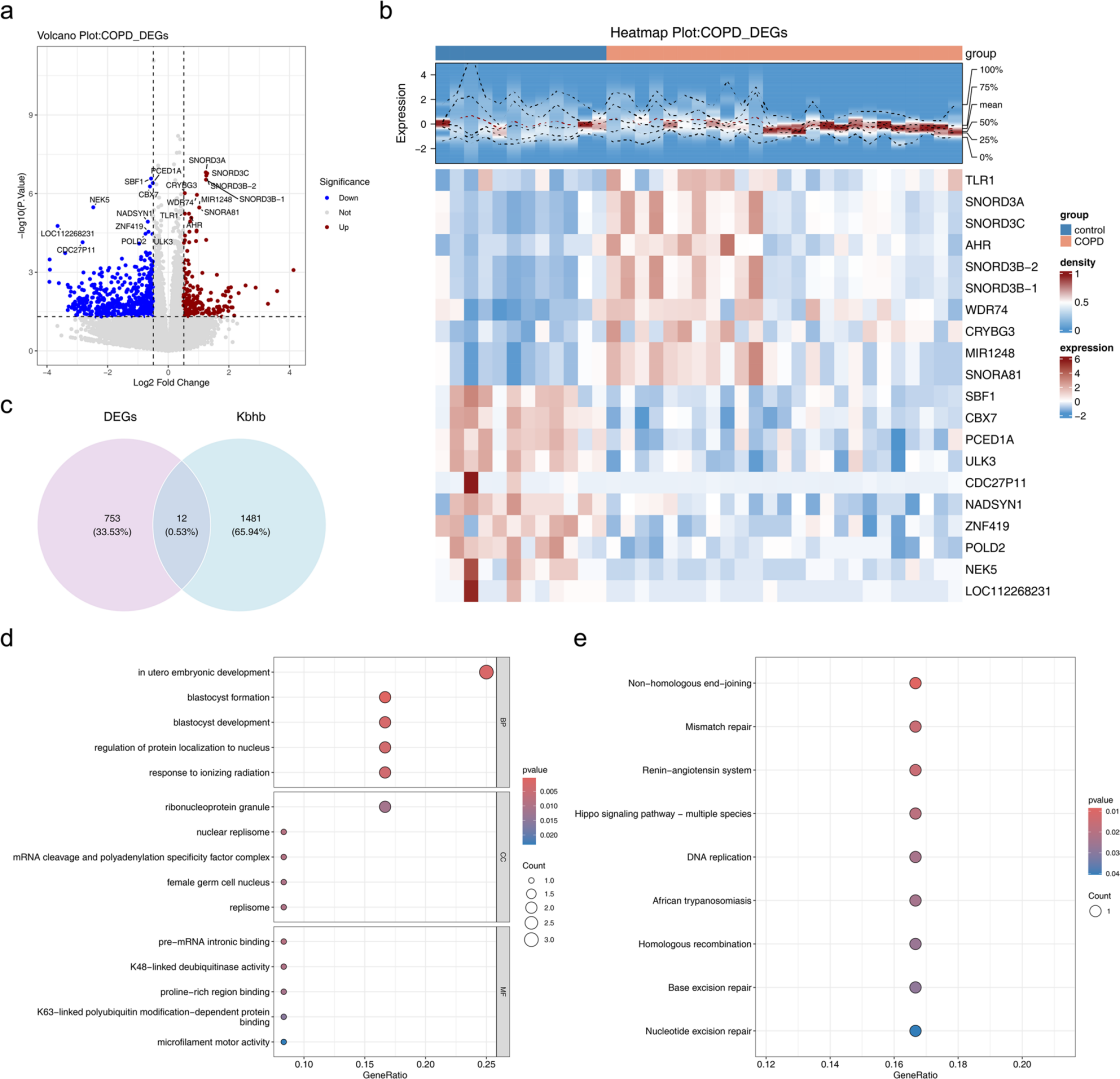
Identification of candidate genes associated with Kbhb between COPD and control in the GSE248493 dataset. **a** Volcano plot depicting DEGs between COPD and control samples. The x-axis represents log₂FC, and the y-axis shows -log₁₀(P value). Genes with |log₂FC| > 0.5 and *P* < 0.05 are highlighted in red (upregulated) and blue (downregulated). Dashed vertical lines indicate the fold change threshold. **b** Heat map of DEGs between the COPD and healthy control samples in the GSE248493 dataset. Rows represent genes, columns represent samples. Color intensity indicates normalized expression values. **c** Venn diagram of overlapping genes between DEGs and Kbhb-RGs. **d** Bubble chart of GO enrichment analysis. The horizontal axis represents gene proportions, while the vertical axis lists the BP, CC, and MF classifications. Bubble size indicates the number of enriched genes within each classification, with colour intensity denoting statistical significance (P-value). **e** Bublle chart of KEGG enrichment analysis

Differential expression analysis showed that there were 765 DEGs between the COPD group and the control group. Among them, 210 genes were identified as upregulated and 555 as downregulated in the COPD group (Fig. 1a-b). Moreover, a total of 12 shared genes between DEGs and Kbhb-RGs were identified and selected as candidate genes (Fig. 1c). The enrichment analysis of the 12 candidate genes revealed associations with 158 GO terms, including 132 BPs, 8 MFs, and 18 CCs (*P* < 0.05) (Additional file 3). These terms encompassed a wide range of functions such as in utero embryonic development, ribonucleoprotein granule, and pre-mRNA intronic binding, among others (Fig. 1d). Moreover, the KEGG enrichment analysis showed that a total of 9 KEGG pathways were enriched, including non-homologous end-joining, mismatch repair, renin-angiotensin system, DNA replication, homologous recombination, and base excision repair (*P* < 0.05) (Fig. 1e).

### POLD2 and OTUD7B were identified as biomarkers

**Fig. 2:**
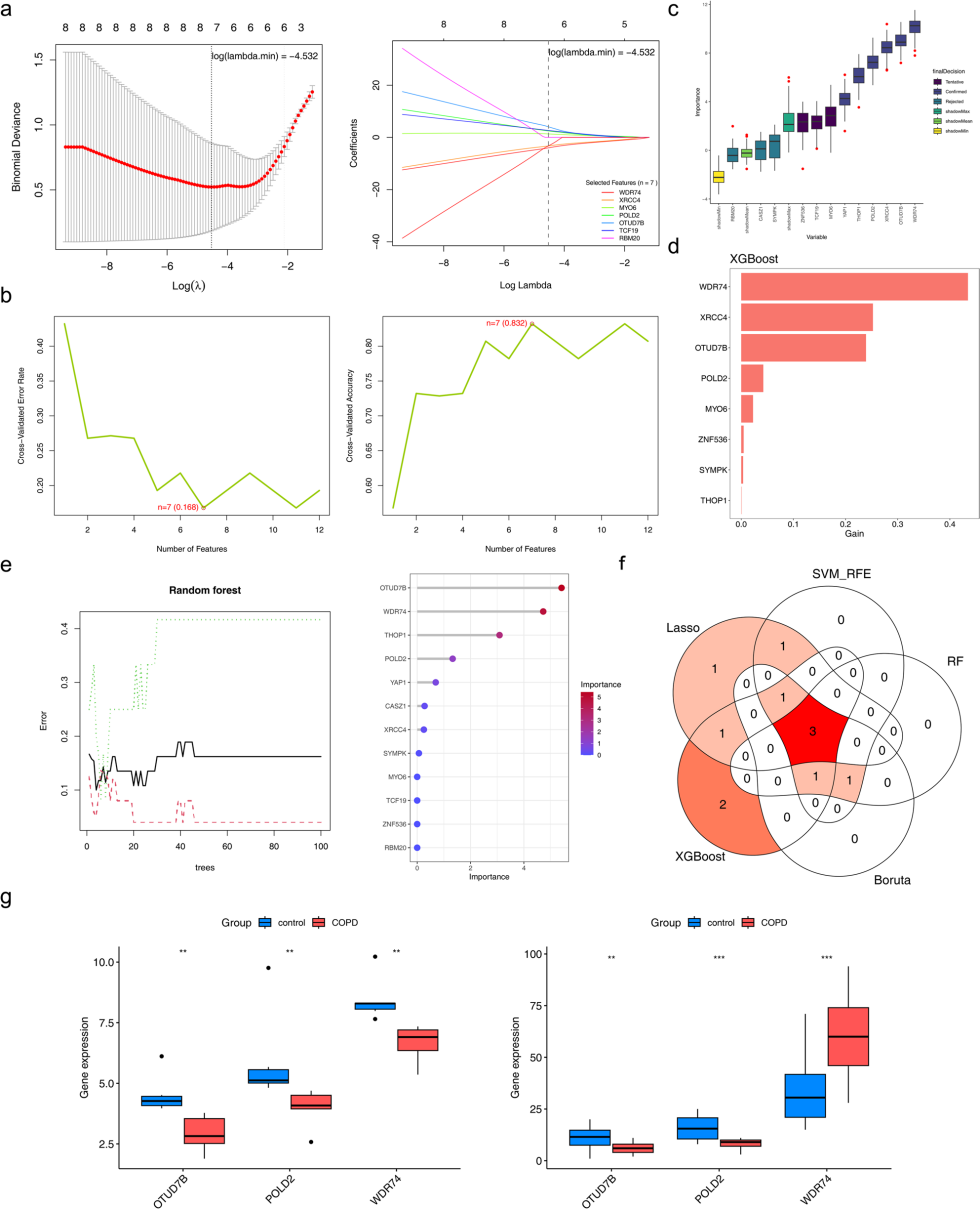
Identification of two biomarkers by five machine learning algorithms. **a** Left panel: Cross-validation plot for LASSO regression, illustrating the relationship between Binomial Deviance and log(λ). The vertical dashed line indicates the optimal parameter λ (λ.min). Right panel: Coefficient path plot showing how gene coefficients vary with log(λ). The vertical dashed line corresponds to λ.min. **b** Error rate curve and accuracy rate curve of SVM-RFE model. The red dot indicates the optimal number of features (7) with the highest accuracy. **c** Boruta feature importance box plot, displaying the score distributions for confirmed features (blue), tentative features (purple), and rejected features (cyan). **d** Bar chart of feature importance for XGBoot model. **e** Left figure: Error rate curve of the random forest model as the number of trees varies. The horizontal axis represents the number of trees, while the vertical axis denotes the error rate. Right figure: Feature importance bubble chart. The horizontal axis displays the importance scores of features, while the vertical axis lists the names of each feature. **f** Venn diagram of five machine learning algorithms. **g** Boxplots showing expression levels of POLD2, WDR74, and OTUD7B in training (GSE248493) and validation (GSE94916) datasets. P values were calculated using Wilcoxon rank-sum test. **P < 0.01, ***P < 0.001

For the results of LASSO algorithm, when the minimum lambda value was 0.01, 7 LASSO genes were screened from the candidate genes (Fig. 2a). When applying the SVM-RFE algorithm, the model demonstrated the highest prediction accuracy with an optimal variable count of 7. This configuration enabled the selection of 7 SVM-RFE genes from the candidate genes (Fig. 2b). Through the Boruta model, 6 Boruta genes were selected from the 12 candidate targets (Fig. 2c). An evaluation of the feature importance assessment results from the XGBoost algorithm was conducted, leading to the selection of 6 XGBoost genes (Fig. 2d). When the RF model was configured, the resulting model error rate reached its minimum, and the top 5 RF genes were identified based on their importance (Fig. 2e). Subsequently, an overlap analysis was conducted on the genes identified by the aforementioned machine learning algorithms. This analysis ultimately identified WDR74, POLD2, and OTUD7B as candidate biomarkers (Fig. 2f), as they were the common genes across all algorithms. According to the GSE248493 and GSE94916 datasets, POLD2 and OTUD7B exhibited markedly low expression levels in the COPD group (Fig. 2g). Consequently, 2 biomarkers—POLD2 and OTUD7B—were selected for subsequent analysis.

### The nomogram model based on the biomarkers was found to be a more effective tool for predicting COPD

**Fig. 3:**
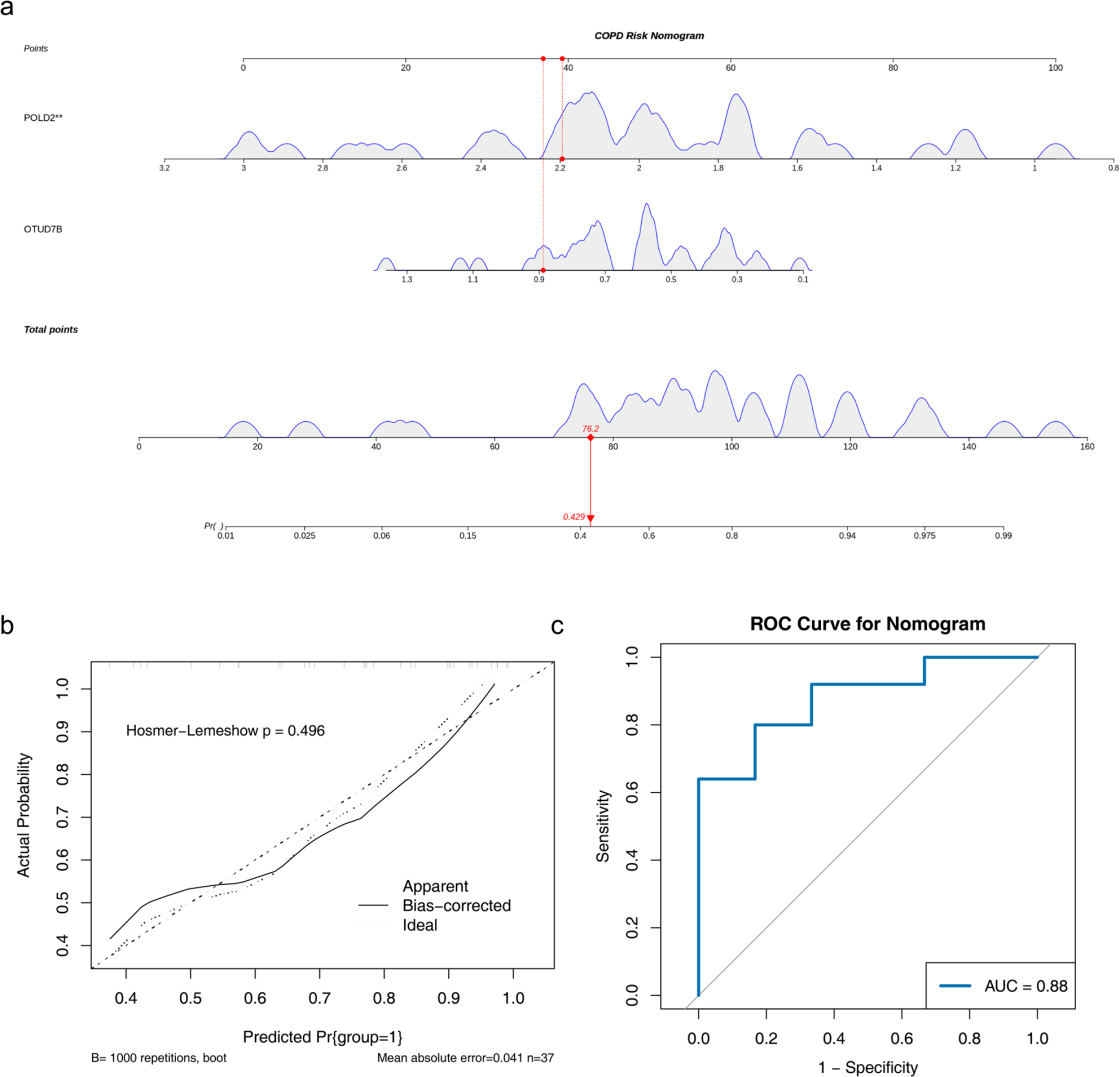
Diagnostic value of the two biomarkers. **a** Nomogram for predicting COPD risk based on POLD2 and OTUD7B expression levels. Points are assigned for each biomarker, and total points correspond to predicted probability of COPD. **b** Calibration curve, with the horizontal axis representing predicted probability and the vertical axis representing actual probability. The dotted diagonal line indicates perfect prediction. Hosmer-Lemeshow test P-value = 0.496. **c** ROC curve of nomogram. The model’s classification capability is assessed by plotting sensitivity and 1-specificity at different thresholds

A nomogram based on the biomarkers was constructed to predict the risk of COPD (Fig. 3a). It was evident from the calibration curve that there was a minimal discrepancy between the actual and predicted risks of COPD (*P* = 0.496) (Fig. 3b). Moreover, the nomogram demonstrated an AUC value of 0.88 (Accuracy: 0.81, Sensitivity: 0.82, Specificity: 0.78), indicating a high degree of accuracy (Fig. 3c). These findings confirmed that the nomogram demonstrates high predictive accuracy for COPD risk.

### Exploring the functions of the biomarkers

**Fig. 4:**
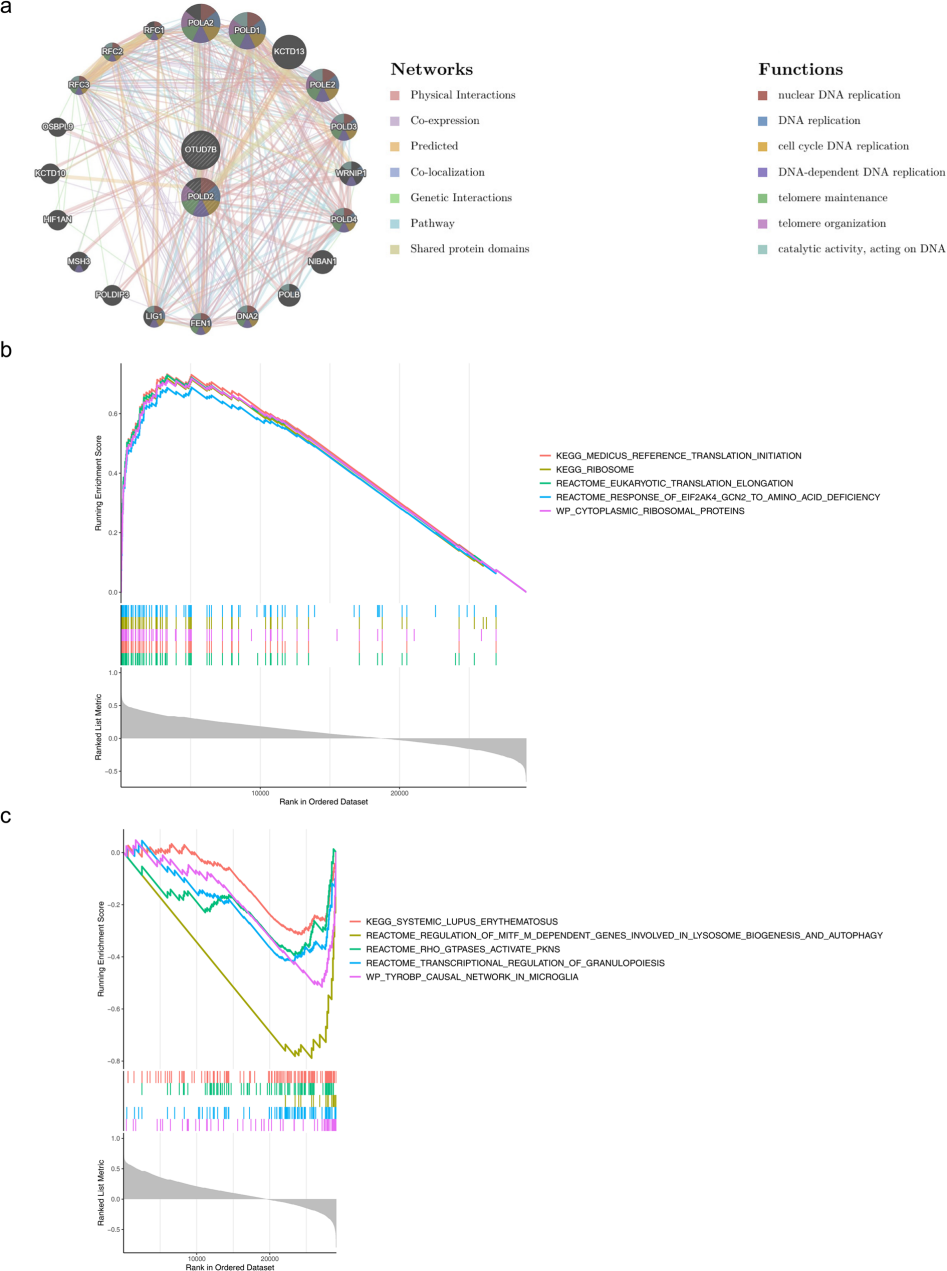
Functional analysis of two biomarkers. **a** Network diagram of GeneMANIA analysis. Nodes in the network diagram represent genes, while the lines connecting nodes denote different types of interactions. **b** GSEA enrichment plot for POLD2. **c** GSEA enrichment plot for OTUD7B. The x-axis represents the ranked position of genes in the dataset, and the y-axis represents the Running Enrichment Score

In the GeneMANIA network, biomarkers interacted with 20 genes, such as POLA2, RFC1, and FEN1, and they were involved in functions such as nuclear DNA replication, DNA replication, and cell cycle DNA replication (Fig. 4a). Subsequently, GSEA revealed that 115 and 60 pathways were enriched with respect to the expression of POLD2 and OTUD7B, respectively (Additional file 4). Notably, POLD2 was remarkably enriched in cytoplasmic ribosomal proteins, ribosome, and eukaryotic translation elongation, while OTUD7B was remarkably enriched in systemic lupus erythematosus, transcriptional regulation of granulopoiesis, and TYROBP causal network in microglia (Fig. 4b-c). These results suggested that COPD was associated with significant alterations in transcriptional regulation and translation processes, which might correlate with the characteristics of COPD.

### There was a strong correlation between biomarkers and immune cells in COPD

**Fig. 5:**
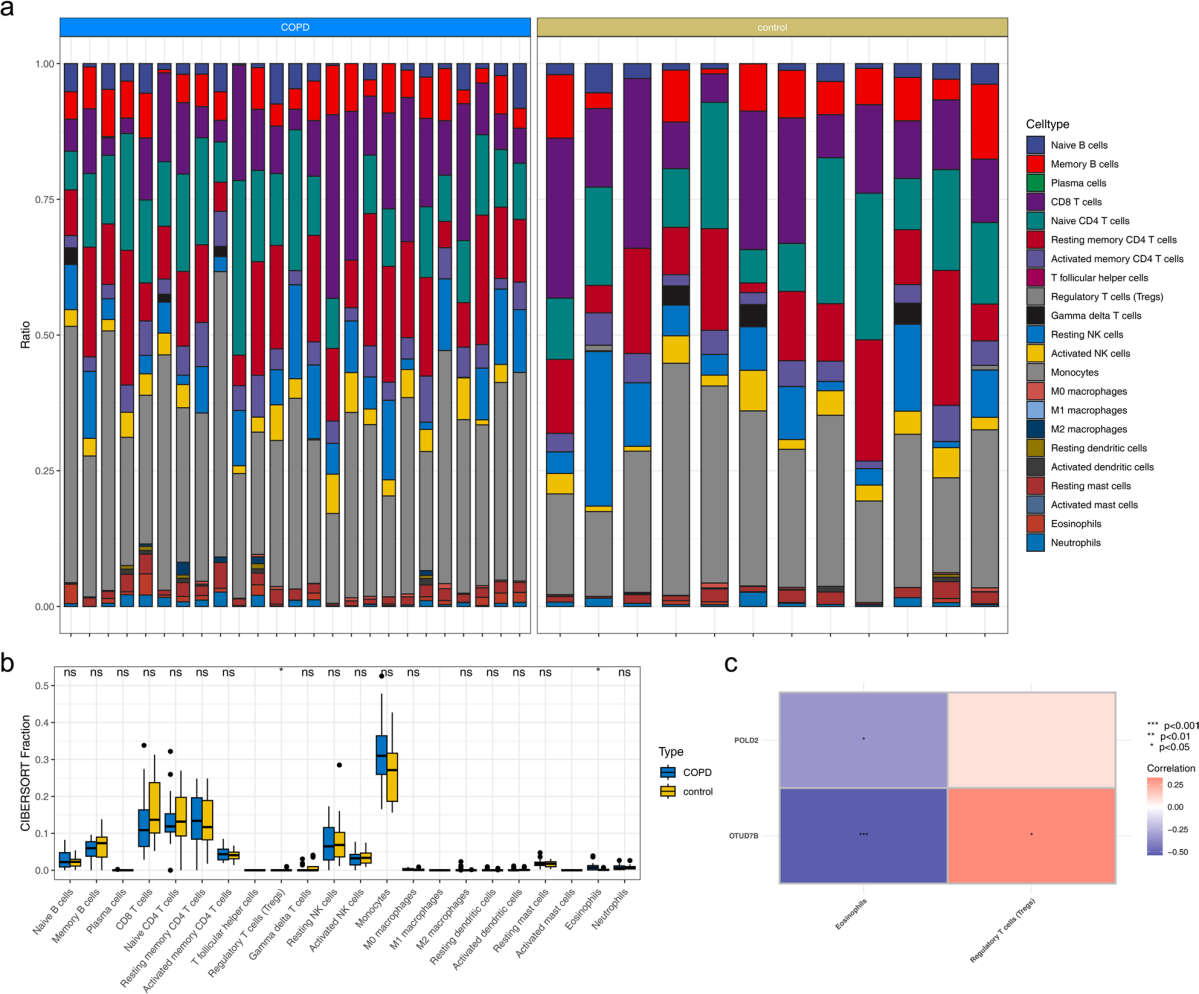
The correlation between biomarkers and immune cells in COPD. **a** Stacked bar chart showing relative proportions of 22 immune cell types in COPD and control samples, estimated by CIBERSORT. **b** Boxplots comparing immune cell infiltration between COPD and control groups. Only eosinophils and Tregs showed significant differences (Wilcoxon test, *P* < 0.05). **c** Correlation diagram of biomarkers and immune cells. Red indicates a positive correlation, blue indicates a negative correlation. *P < 0.05, ***P < 0.001

The onset of disease is frequently associated with the function of the immune system. Consequently, the examination of immune cell distribution facilitates a more comprehensive investigation of disease pathogenesis. The dispersion of 22 immune cell types in the COPD and control groups was illustrated, which displayed varying degrees of infiltration for each immune cell type (Fig. 5a). A total of 2 immune cell types exhibited marked variations in infiltration between the 2 groups (*P* < 0.05) (Fig. 5b). Of these, eosinophils showed significantly higher levels in the COPD group compared with the control group. In addition, Regulatory T cells demonstrated a significant positive correlation with OTUD7B (*r* > 0.3, *P* < 0.05). Eosinophils were significantly adversely linked to POLD2 and OTUD7B (*r* < -0.3, *P* < 0.05) (Fig. 5c). These findings provided significant insights into the molecular mechanisms involved in the pathogenesis of COPD.

### Biomarkers were targeted by miRNAs and TFs

**Fig. 6:**
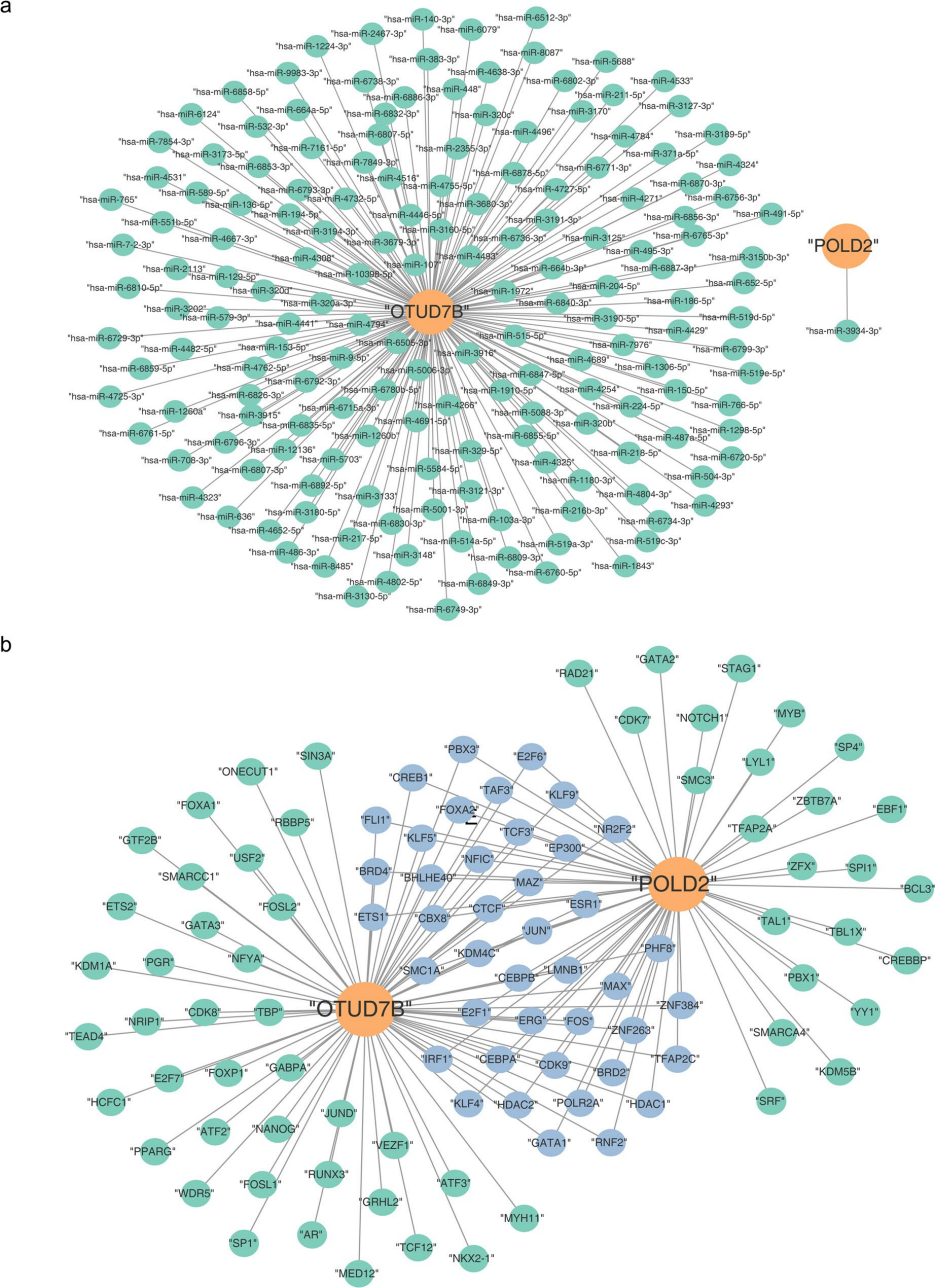
Molecular regulatory networks of two biomarkers. **a** miRNA-mRNA interaction regulatory network. Orange dots represent mRNA, green dots denote miRNAs, and connecting lines indicate interactions between them. **b** TF-mRNA network diagram. Orange dots denote mRNA, green dots denote predicted TFs, and blue dots denote common TFs

In this study, 169 key miRNAs targeting the biomarkers were identified from the databases (Fig. 6a). Specifically, 168 miRNAs were found to target OTUD7B, while 1 miRNA targeted POLD2. These miRNAs were suggested to play crucial regulatory roles in the expression of POLD2 and OTUD7B, potentially influencing their downstream functions in the disease context. Additionally, some TFs targeting biomarkers were identified. In particular, nearly 39 TFs—including PBX3, CREB1, and E2F6—were found to simultaneously regulate the expression of these two biomarkers (Fig. 6b). This co-targeting indicated that these TFs might have coordinated roles in regulating POLD2 and OTUD7B expression, highlighting their potential importance in the regulatory network underlying disease pathogenesis.

### Biomarkers exhibited strong binding affinity with estradiol

**Fig. 7:**
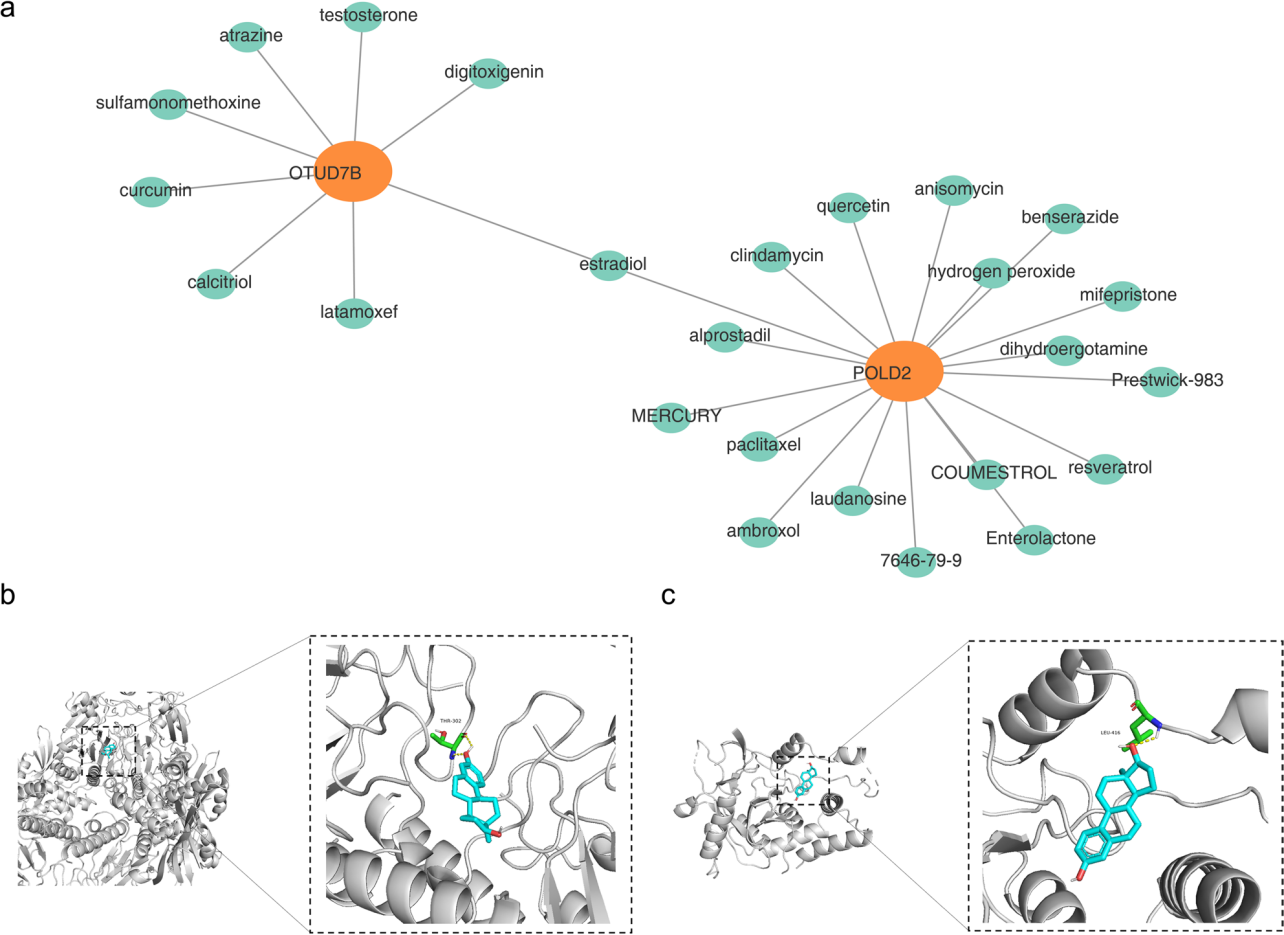
Drug prediction and molecular docking. **a** Drug-biomarker interaction network. Orange dots denote biomarkers, while green dots denote drugs. **b** Molecular docking model of POLD2 with estradiol. **c** Molecular docking model of OTUD7B with estradiol

Potential drugs targeting the biomarkers were retrieved from the Drug-Gene Interaction Database, as shown in Fig. 7a. These included 18 drugs that targeted POLD2, and 8 drugs that targeted OTUD7B. Notably, only 1 drug that jointly acted on biomarkers was screened, namely estradiol. According to the molecular docking results, the docking binding energies of the POLD2-estradiol and OTUD7B-estradiol were all less than − 5 kcal/mol (Table 1). The specific binding modes of the POLD2-estradiol and OTUD7B-estradiol complexes were shown in Fig. 7b-c. This provides an initial computational clue for exploring whether estradiol or its analogs could modulate these two targets and thereby influence COPD progression.

**Table 1:** Binding energy of biomarkers and small molecular compounds of drugs

Ligand	MF	Proteins	Binding Afinity (keal/mol)	Interacting amino acids
estradiol	C18H24O2	OTUD7B	-8.1	LEU-416
		POLD2	-8.5	THR-302

### ScRNA-seq data annotation revealed 17 cell types

**Fig. 8:**
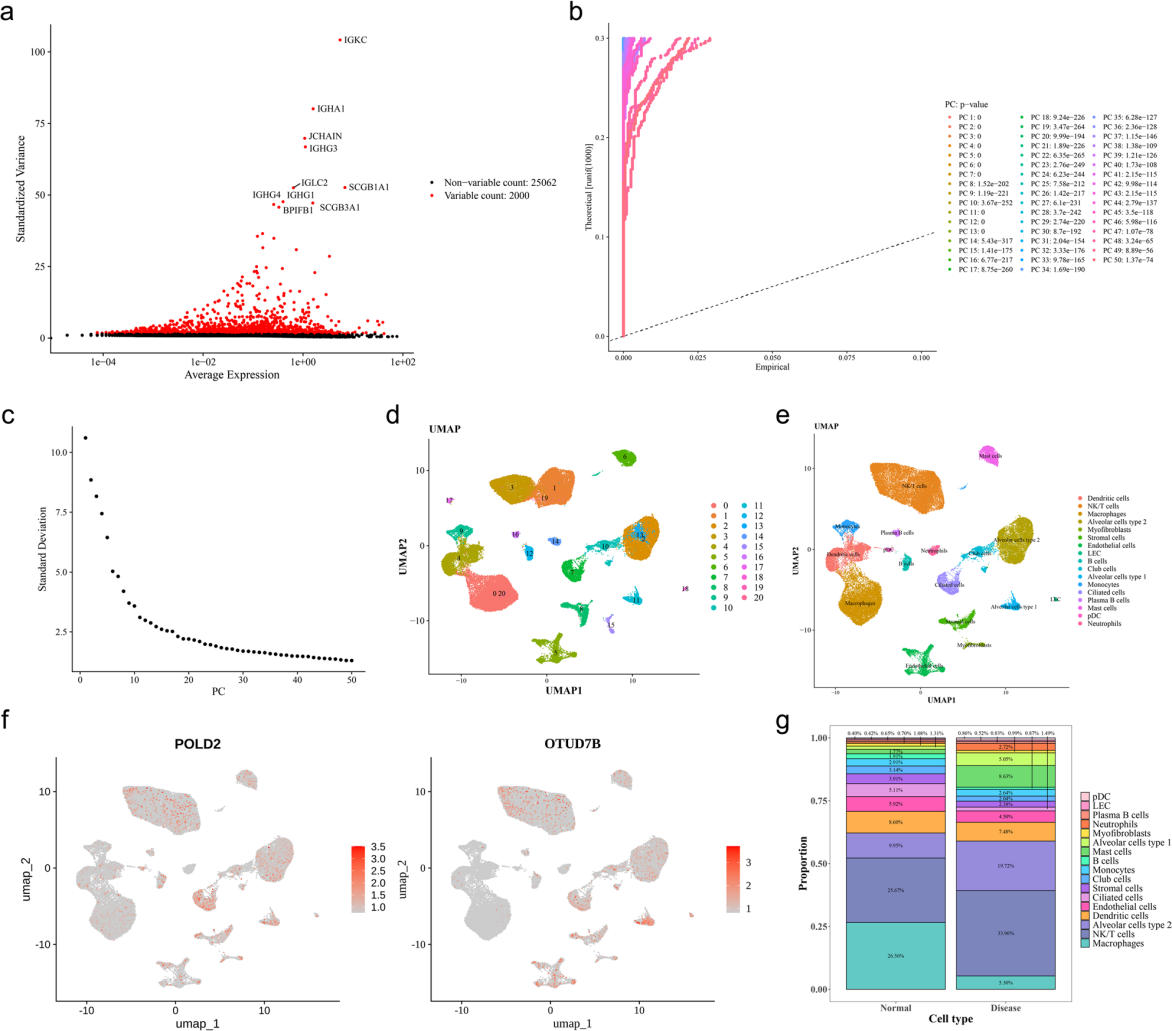
ScRNA-seq data annotation revealed 17 cell types. **a** Visualization of highly variable genes. Red dots indicate highly variable genes, while black dots represent non-highly variable genes. **b** Jackstraw plot for PCA. The horizontal axis shows empirical values calculated by the Jackstraw function, the vertical axis displays computed theoretical values, and the legend on the right indicates the P-value for each principal component. **c** Number of available dimensions in the fragment plot. The horizontal axis represents the number of PCs, while the vertical axis shows the standard deviation. **d** UMAP plot of cell clusters. The x-axis represents the first principal component axis after UMAP dimensionality reduction, while the y-axis represents the second principal component axis. Points of different colors denote distinct cell clusters. **e** UMAP plot of annotation results for 17 cell types across all samples. **f** Expression distribution of POLD2 and OTUD7B across different cell types. Red dots indicate biomarker expression in the corresponding cell type. **g** Proportional distribution diagram of different cell types

In GSE171541, there were 6,459 cells and 17,966 genes that met the criteria (Additional file 5). Subsequently, the analysis generated 2,000 highly variable genes with the largest variance, such as IGKC (Fig. 8a). Among the highly variable genes, the top 20 PCs) contributed the most to the variation (Fig. 8b-c). Subsequently, 21 cell clusters were acquireed by clustering depending on the top 20 PCs (Fig. 8d). These 21 cell clusters were classified into 17 cell types, including Dendritic cells, Natural Killer T (NK/T) cells, Macrophages, Alveolar cells type 2, Myofibroblasts, Stromal cells, Endothelial cells, Lymphatic Endothelial Cells (LEC), B cells, Club cells, Alveolar cells type 1, Monocytes, Ciliated cells, Plasma B cells, Mast cells, Plasmacytoid Dendritic Cells (pDC), and Neutrophils (Fig. 8e). Furthermore, the expression distribution of biomarkers across different cell types was analysed. Results revealed that POLD2 and OTUD7B were detected in all annotated cell types. Cell types exhibiting relatively high expression levels included NK/T cells, Ciliated cells, Alveolar cells type 1, Alveolar cells type 2, and Mast cells (Fig. 8f). By comparing the cellular composition between the disease group and the control group, a significant difference in the proportion of mast cells was observed between the two groups (Fig. 8g, Additional file 6). In conjunction with previous literature reporting the role of mast cells in COPD [32], the findings of this study suggest that mast cells may play a significant role in the pathological mechanisms of COPD, warranting further functional investigations in future studies.

### Biomarkers were significantly downregulated in clinical samples from COPD patients

**Fig. 9:**
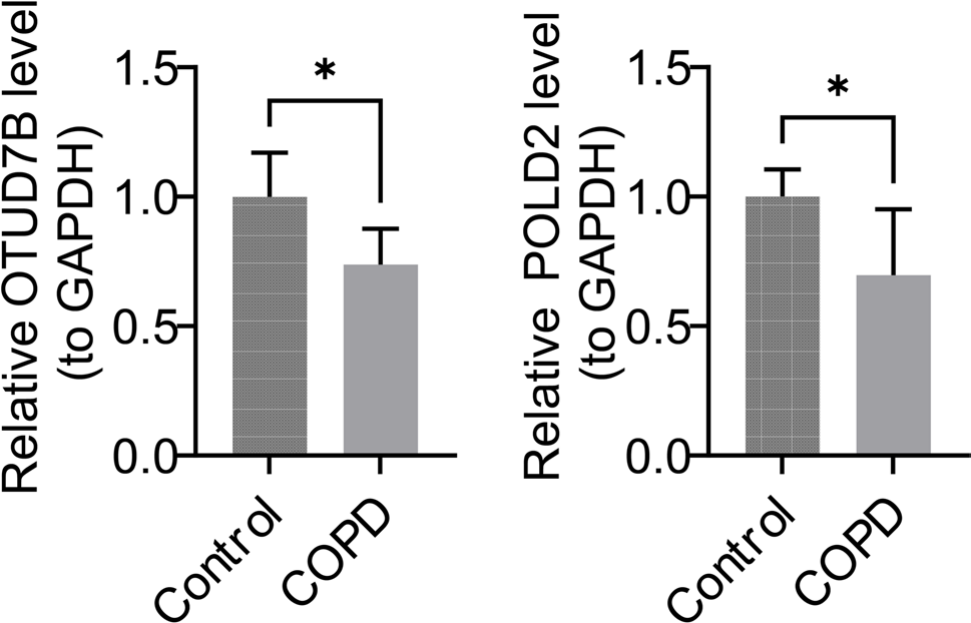
Validation of biomarker expression by RT-qPCR. Boxplots showing relative mRNA expression levels of POLD2 and OTUD7B in PBMC samples from COPD patients (*n* = 5) and healthy controls (*n* = 5). Data are normalized to GAPDH and presented as 2^−ΔΔCT^. P values were calculated using unpaired t-test. *P < 0.05

To further validate the expression of biomarkers, we conducted RT-qPCR experiments using clinical samples. The results presented in Fig. 8 demonstrated a substantial decrease in the expression levels of POLD2 and OTUD7B in the COPD group compared to the control group (*P* < 0.05). These findings were consistent with our bioinformatics analyses, thereby providing additional support for the hypothesis that Kbhb significantly influenced the progression of COPD (Fig. 9).

## Discussion

Despite substantial advances in diagnostic and therapeutic strategies over the past decade, COPD remains the third leading cause of death worldwide [33–35]. The lack of reliable biomarkers continues to hinder early disease identification [33–35]. Recent studies have implicated Kbhb in the pathogenesis of lung diseases [8], suggesting that Kbhb-related genes may serve as potential biomarkers for early intervention. In this study, we identified POLD2 and OTUD7B as differentially expressed, Kbhb-related biomarkers in COPD through integrative bioinformatics analysis of GEO transcriptomic data. RT-qPCR validation confirmed downregulated expression of both genes in COPD patients. We further explored potential therapeutic targets and molecular regulatory mechanisms. These findings provide novel insights for COPD diagnosis and management.

POLD2 (DNA polymerase delta 2) is essential for DNA replication and repair [36]. DNA polymerase delta participates in the elongation phase of DNA synthesis, ensuring accurate and efficient replication, while also facilitating repair of damaged DNA to maintain genomic stability [37, 38]. Reduced POLD2 expression or function can impair DNA damage repair, triggering inflammatory responses through activation of pathways such as cGAS/STING/NF-κB [39]. We hypothesize that POLD2 may modulate DNA damage-induced inflammation in COPD through its repair function, although further research is needed to elucidate the precise mechanisms.

Chronic exposure to toxic stimuli such as cigarette smoke can damage lung cell DNA. Our study identified downregulated POLD2 expression in COPD, suggesting it may be subject to Kbhb-mediated regulation. Aberrant Kbhb modification of POLD2 could impair its repair capacity, leading to accumulation of DNA damage and potentially promoting alveolar epithelial cell death [5, 40]. Increased alveolar epithelial cell death can disrupt alveolar architecture, contributing to emphysema development [41, 42]—a hallmark of COPD. Thus, the Kbhb-POLD2 axis may represent a novel mechanistic link connecting metabolic dysregulation, DNA damage response, cell death, and emphysema pathogenesis. Functional studies are warranted to establish causal relationships within this proposed mechanism.

Ovarian tumor deubiquitinase 7B (OTUD7B) is a p53-targeting deubiquitinase belonging to the OTU superfamily. This multifunctional enzyme contains a deubiquitinase catalytic domain, a ubiquitin-associated domain, and a zinc finger domain, and has been implicated in cell cycle regulation, neural progenitor development, inflammatory responses, mucosal immunity, and tumorigenesis through substrate-specific deubiquitination [43–46]. OTUD7B suppresses lung cancer cell invasion and migration by deubiquitinating TRAF3, thereby inhibiting non-canonical NF-κB signaling [47]. NF-κB plays a central role in inflammatory regulation. Sustained inflammatory stimulation in COPD activates NF-κB, driving release of pro-inflammatory cytokines including TNF-α and IL-6 [48–50]. OTUD7B may similarly modulate COPD inflammation through NF-κB pathway regulation [44, 51]. Additionally, Kbhb can modify histones, alter chromatin states, influence inflammatory gene transcription, and promote COPD inflammatory progression [52]. These findings suggest that OTUD7B-mediated deubiquitination and Kbhb-mediated histone modification may cooperatively regulate inflammatory factor expression in COPD pathogenesis.

Based on our findings and current literature, we propose the following testable hypothesis: Elevated β-hydroxybutyrate may serve as a metabolic co-substrate in the COPD microenvironment [4], promoting specific histone Kbhb modifications (e.g., H3K9kbhb) at the POLD2 and OTUD7B promoter regions [8]. Such epigenetic alterations may suppress transcription through chromatin remodeling or recruitment of specific reader proteins, consistent with the observed downregulation in COPD samples. POLD2 downregulation could impair DNA repair, leading to genomic instability and alveolar epithelial cell apoptosis [40–42]. OTUD7B downregulation may activate pro-inflammatory pathways such as NF-κB through altered ubiquitination of substrates including TRAF3 [47]. Coordinated dysregulation of these pathways may contribute to characteristic COPD pathologies, including alveolar destruction and chronic airway inflammation. Future studies should validate this regulatory axis using ChIP-seq, reporter assays, and site-specific epigenetic editing in relevant model systems.

GSEA revealed enrichment of POLD2 and OTUD7B in pathways related to granulopoiesis regulation, ribosome function, and eukaryotic translation elongation. Ribosomes constitute the primary machinery for protein synthesis, with ribosome biogenesis involving rRNA transcription, processing, modification, assembly, and export [53, 54]. COPD patients exhibit reduced 12 S/16S rRNA ratios, indicative of mitochondrial ribosomal stress and dysfunction [55]. Mitochondria serve as cellular energy generators, with mitochondrial ribosomes playing essential roles in maintaining oxidative phosphorylation capacity. Impaired mitochondrial ribosome function can disrupt respiratory chain protein synthesis, leading to energy deficiency and cellular dysfunction [41, 56]. Such bioenergetic impairment may contribute to exercise intolerance and reduced physical performance in COPD patients, negatively impacting quality of life and daily activities [56–58]. We hypothesize that downregulation of POLD2 and OTUD7B may impair mitochondrial function through ribosomal dysregulation, contributing to COPD pathophysiology. This hypothesis requires experimental validation.

Immune infiltration analysis revealed negative correlations between both biomarkers and eosinophil levels. These findings suggest that POLD2 and OTUD7B may modulate the COPD immune microenvironment through effects on eosinophil abundance or function. Elevated blood eosinophil counts in COPD are associated with type 2 inflammation [59]. Eosinophils are activated by cytokines including IL-3, IL-5, and GM-CSF, and subsequently migrate to inflamed tissues [60, 61]. Anti-IL-5 monoclonal antibodies, which target eosinophil activation, effectively attenuate type 2 inflammation [62]. Eosinophilic inflammation occurs during both acute exacerbations and stable phases of COPD [63], suggesting that POLD2 and OTUD7B may serve as biomarkers for monitoring type 2 inflammatory responses and evaluating biologic therapy efficacy.

We acknowledge that our immune infiltration analysis was based on PBMC data, which excludes granulocytes; thus, circulating monocyte profiles may not fully reflect lung macrophage polarization. Consequently, our findings primarily represent systemic rather than local pulmonary immune features. Nevertheless, COPD is a systemic disease with peripheral immune states reflecting pulmonary pathology [64]. Moreover, our objective was to identify peripheral blood biomarkers for non-invasive diagnostic applications, for which PBMC analysis is appropriate. The observed associations between POLD2/OTUD7B and immune cells suggest roles in systemic immune regulation in COPD. Future studies incorporating lung tissue samples are needed to elucidate local mechanisms.

Our drug prediction analysis identified estradiol as a potential dual-targeting compound for POLD2 and OTUD7B, with favorable binding energies. Estradiol has demonstrated protective effects against lung injury in traumatic contexts [65]. Epidemiologically, estradiol levels are inversely associated with COPD risk (OR 0.794, 95% CI 0.688–0.915, *p* = 0.005) [65]. Mechanistically, estradiol modulates estrogen receptor alpha expression and NF-κB activity, ameliorating COPD-like phenotypes in animal models [66]. We hypothesize that estradiol may exert therapeutic effects in COPD partially through modulation of POLD2 and OTUD7B, although this mechanism requires experimental validation.

In conclusion, we identified POLD2 and OTUD7B as Kbhb-related biomarkers in COPD that appear to modulate immune cell function and disease pathogenesis. These findings provide a foundation for understanding COPD pathophysiology and improving clinical management. However, several limitations should be acknowledged. First, the sample size was relatively small. Although regularization, cross-validation, and multi-algorithm consensus approaches were employed to enhance robustness, validation in larger, multi-center cohorts is needed. Second, as a bioinformatics screening study, the functional mechanisms of POLD2 and OTUD7B in COPD remain to be elucidated. Future studies will systematically investigate POLD2-mediated DNA repair and OTUD7B-mediated NF-κB regulation in relevant cell models. Third, Kbhb modifications were not assessed at the genomic or proteomic levels. Future work will employ ChIP-seq and proteomic approaches to characterize the Kbhb landscape in COPD and quantify promoter modifications at these loci, thereby comprehensively elucidating the epigenetic regulatory network.

## Conclusions

This study identified POLD2 and OTUD7B as novel Kbhb-related biomarkers for COPD. Validated in clinical samples, their downregulation is significantly associated with disease progression. Functional analyses implicated their roles in DNA repair, inflammatory response via the NF-κB pathway, and immune regulation, particularly eosinophil infiltration. Furthermore, molecular docking suggested estradiol as a potential therapeutic agent targeting these biomarkers. These findings provide new insights into the epigenetic mechanisms of COPD and offer promising targets for early diagnosis and future therapeutic intervention, although further experimental validation is required.

## Supplementary Information

Additional file 1. The list of 1,493 Kbhb-RGs.

Additional file 2. Primer sequences of RT-qPCR.

Additional file 3. The results of GO enrichment analysis for candidate genes.

Additional file 4. The results of GSEA for POLD2 and OTUD7B.

Additional file 5. Distribution plots of nFeature_RNA, nCount_RNA, and percent.mt before and after quality control.

Additional file 6. Violin plot showing the proportion differences of different cell types in the disease group versus the control group.

## Data Availability

The data that support the findings of this study are openly available in the NCBI GEO repository at https://www.ncbi.nlm.nih.gov/geo/, reference number GSE248493, GSE94916 and GSE171541.

## Abbreviations

COPD: Chronic Obstructive Pulmonary Disease
Kbhb: Lysine β-hydroxybutyrylation
Kbhb-RGs: Kbhb-related genes
DEGs: Differentially expressed genes
RT-qPCR: Reverse transcription quantitative polymerase chain reaction
CoA: Coenzyme A
BHB: β-hydroxybutyrate
GEO: Gene Expression Omnibus
PBMC: Peripheral blood mononuclear cell
FC: Fold Change
GO: Gene Ontology
MF: Molecular function
BP: Biological process
CC: Cellular component
KEGG: Kyoto Encyclopedia of Genes and Genomes
LASSO: Least Absolute Shrinkage and Selection Operator
SVM-RFE: Support Vector Machine-Recursive Feature Elimination
XGBoost: eXtreme Gradient Boosting
RF: Random Forest
HL: Hosmer-Lemeshow
ROC: Receiver Operating Characteristic
AUC: Area under the curve
GeneMANIA: Gene Multiple Association Network Integration Algorithm
GSEA: Gene set enrichment analysis
MiRNAs: MicroRNAs
TFs: Transcription Factors
POLD2: DNA polymerase delta 2
OTUD7B: Ovarian tumor ubiquitin-degrading enzyme 7B
IL: Interleukins
