# Supplementary figures and images for: Identification and validation of biomarkers related to lysine β-hydroxybutyrylation in chronic obstructive pulmonary disease based on transcriptomics data
Source: Hereditas. 2026 Apr 24;163:74. doi: 10.1186/s41065-026-00682-x (PMC13245097; doi:10.1186/s41065-026-00682-x)

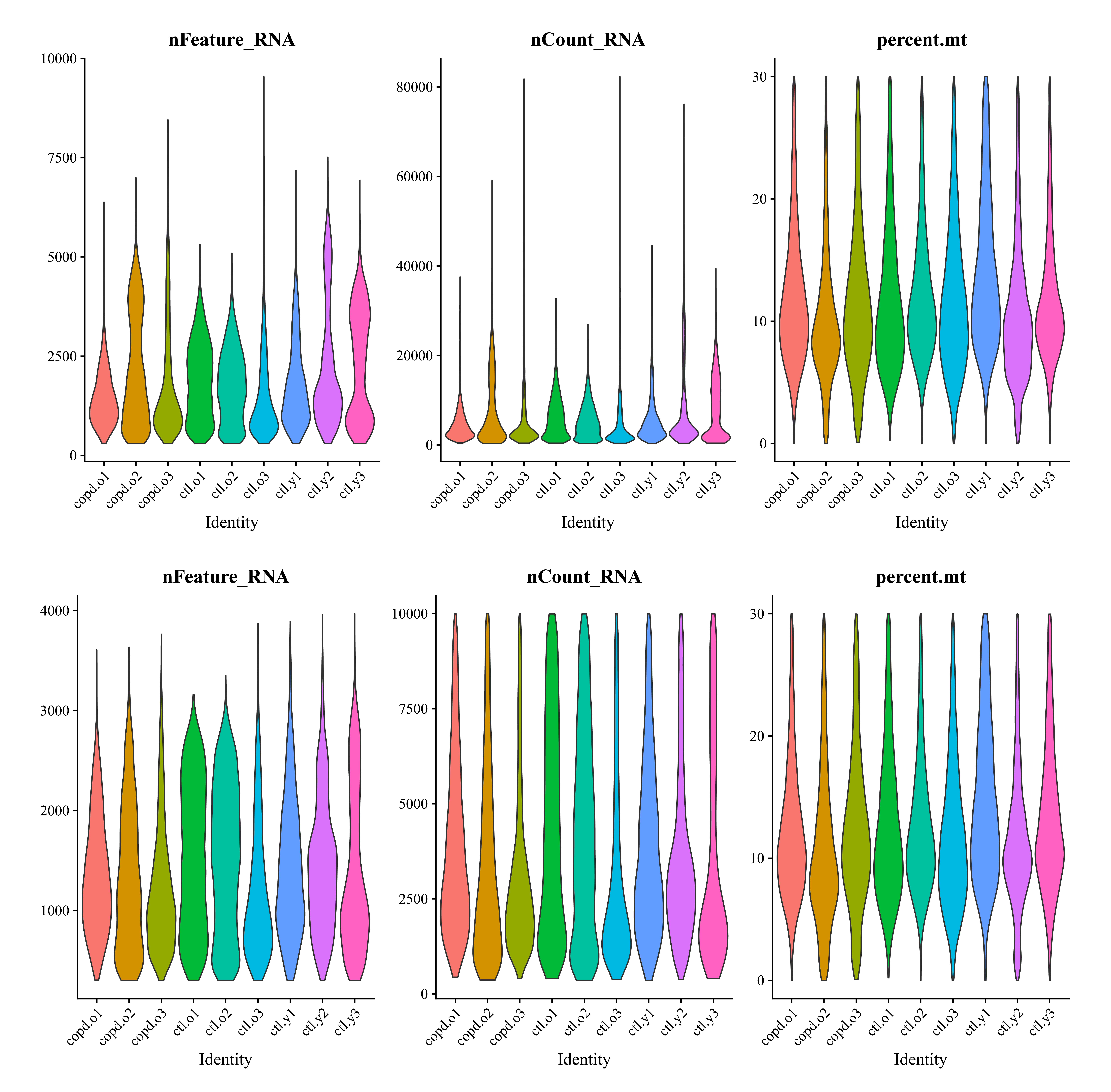

Supplement: Supplementary file 5 — Additional file 5. Distribution plots of nFeature_RNA, nCount_RNA, and percent.mt before and after quality control. [file 41065_2026_682_MOESM5_ESM.tif]

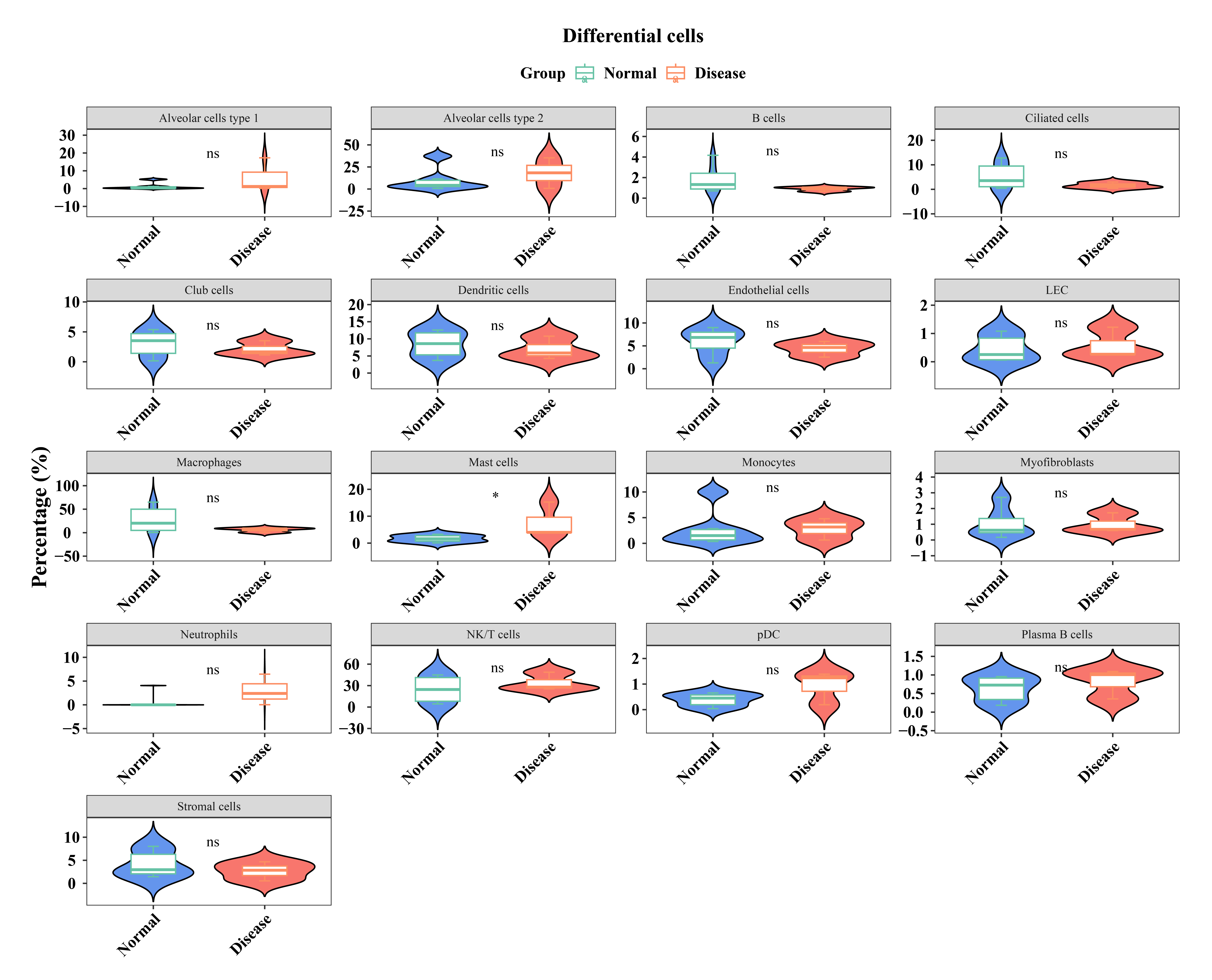

Supplement: Supplementary file 6 — Additional file 6. Violin plot showing the proportion differences of different cell types in the disease group versus the control group. [file 41065_2026_682_MOESM6_ESM.tif]
